# What was really wrong (and right) with vitalism? Methodological naturalism, organicism and immaterialist theories in biology

**DOI:** 10.1007/s40656-026-00719-4

**Published:** 2026-02-24

**Authors:** Sandy C. Boucher

**Affiliations:** School of Humanities, Arts and Social Sciences, University of New England, Armidale, NSW 2351 Australia

**Keywords:** Methodological naturalism, Vitalism, Organicism, Holism, Reductionism

## Abstract

There has been considerable discussion in recent years of *methodological naturalism* in the sciences: the question of whether science does, or should, operate under the assumption that it may only investigate natural phenomena or offer natural explanations, and what form this assumption does or should take. Most of the discussion has centred around theories such as intelligent design creationism, but in this paper I argue that vitalism in biology—an immaterialist theory of living systems—provides an instructive and under-utilised test-case for methodological naturalism. I focus on three views: intrinsic methodological naturalism, provisional/pragmatic methodological naturalism, and, in particular, the ‘unintelligibility’ view I have previously defended: views which have not, for the most part, been applied to vitalism. The unintelligibility view comes in a metaphysical, and an epistemic form. I suggest that on the epistemic interpretation theories such as vitalism should be understood along the lines of ‘mysterian’ or ‘ignorance’ accounts of consciousness, and that this provides an illuminating account both of vitalism itself, and of its relationship to organicism in biology.

## Introduction

*Methodological naturalism* (MN) with respect to the sciences is the view that science does, or should, only investigate natural phenomena, or offer natural explanations. Recent discussions of MN have focused almost entirely on religiously-motivated views such as intelligent design creationism (IDC). This paper considers MN in the context of a quite different immaterialist theory: vitalism in biology. I shall frame the discussion in terms of three prominent views with respect to MN: intrinsic MN, provisional/pragmatic MN, and, in particular, the ‘unintelligibility view’ I have previously defended (2020). I will suggest that on one plausible reading of the unintelligibility view, the ‘epistemic’ reading, vitalism can be construed as a form of mysterianism, akin to mysterian or ‘ignorance’ accounts of consciousness. This has the potential to furnish us with an illuminating account both of vitalism itself, and of its relationship to *organicism* in biology, I shall argue.

This is not merely of historical interest, since organicism has experienced a notable revival in recent biology and philosophy of biology. Thus, this paper has two aims: to contribute to the debate around MN by considering and assessing some prominent accounts of MN in light of the neglected case of vitalism; and to contribute to the historical and philosophical debate around organicism and its relation to other views, by providing a means by which contemporary organicists can (via the epistemic version of the unintelligibility view) reconsider and possibly re-evaluate vitalism’s contribution to biology, in light of organicist commitments.

The plan of the papers is as follows. In Sect. 2, I summarise the three views concerning MN. In Sect. 3, I consider what the three views would say about the case of vitalism. I give some reasons for thinking the unintelligibility view (on the metaphysical interpretation) fits the case of vitalism better than the other two views, but that this is not decisive. In Sect. 4, I outline the epistemic construal of the unintelligibility view; I argue that it is coherent in itself, and also that there are good grounds for interpreting vitalism in accordance with it. In Sect. 5, I suggest that on this interpretation vitalism has much in common with mysterian or (weaker) ignorance accounts of consciousness. Section 6 is the key section of the paper. It examines the interpretation of vitalism as a view similar to Stoljar’s ignorance view of consciousness (2006). In Sect. 7, I respond to the potential worry that if my analysis of vitalism is correct, it could license a similar reinterpretation and partial rehabilitation of IDC, which would be an unwelcome consequence. Section 8 concludes.

## Methodological naturalism: three views

According to those who defend ‘intrinsic MN’ (IMN), scientific inquiry, by definition, excludes appeal to the supernatural (Haught, 2004; Pennock, 2001, 2011; Ruse, 1982; Scott, 1998, 1999). MN is, on this view, a constitutive assumption or commitment of science: it is *intrinsic* to science that it can only deal in natural causes, processes, and phenomena. This is a demarcation criterion in the traditional sense of a necessary condition for a claim or hypothesis to count as scientific: a claim or hypothesis must only posit or quantify over natural entities, forces, or processes to qualify as scientific. The main reason for this is that supernatural claims are, on this view, intrinsically untestable. A ‘science of the supernatural’ is thus an oxymoron. To put it another way, on this view, if some putatively supernatural claim or hypothesis were a potential part of science, then it would not, in fact, be a supernatural claim or hypothesis, but rather a natural one (it’s no true Scotsman). IMN is explicitly neutral on the truth of ontological naturalism, the view, roughly, that the natural world is all that there is and there are no supernatural entities, forces, or agency. IMN asserts that supernatural claims or hypotheses are unscientific, not that they are false. IMN is thus designed to provide a means of responding to the charge, often made by creationists, that science, or particular scientific theories such as the theory of evolution, unjustifiably presuppose, a priori, a naturalistic metaphysics, rather than allowing the question of whether our metaphysics ought to be naturalistic to be treated as an a posteriori, scientific question, as it ought. Science, and particular scientific theories, do not presuppose any such metaphysical doctrine, according to IMN, but merely the methodological doctrine that science *qua* science is only capable of trafficking in the natural. A scientist who believes in supernatural forces or agency is not acting irrationally, or violating IMN, on this view. She only violates IMN if she holds that whether supernatural forces or agency exist, and what they are like, is a properly scientific question.

According to ‘pragmatic/provisional MN’ (PMN), we ought, today, to be methodological naturalists with respect to science, but IMN is mistaken in thinking that scientific inquiry *necessarily* excludes the supernatural. On this view there is nothing about the supernatural as such, or science as such, that makes it the case that supernatural claims or hypotheses are in principle unable to be investigated using the standard methods of science. MN is justified today, on this view, not by reference to any a priori or constitutive constraint on what counts as scientific, but merely by the poor track record of supernatural theories. Again and again such theories have proven to be less successful than their naturalistic rivals, such that we are entitled to infer that science would do well to avoid repeating past mistakes, and proceed under the assumption that only naturalistic theories are to be taken seriously.^1^ But this is a provisional commitment, and one that is, in principle, capable of being overturned in the future, in the unlikely event that supernatural claims or theories are forced upon us as the only means of accounting for evidence that may arise (for defence and discussion see Dawes, 2011; Smith, 2017; Smith & Dawes, 2018; Fales, 2013; Boudry et al., 2010; Koperski, 2008; McDonald & Tro, 2009).

On the PMN view, the distinction between methodological and ontological naturalism is not as sharp as on the IMN view. We saw that for IMN, supernatural claims or theories are unscientific, but it remains neutral on whether any of them are true. For those who defend PMN on the other hand, supernatural claims and theories have in the past been rejected precisely because they were found by the methods of science to be (probably) untrue. They were rejected in just the way that failed naturalistic theories in science were rejected—the theories of caloric, phlogiston, the ether, Newtonian mechanics, pangenesis and all the rest—i.e., because they were empirically and explanatorily inadequate, and rival theories were superior. And, on the PMN account, the reason science today should not consider supernatural theories is not because it would have no way of testing them, but because it is very likely that they would turn out, like their predecessors, to be false or unjustified, so we shouldn’t waste our time with them. While the claim that past supernatural theories have been unsuccessful, and thus that science today should only admit naturalistic theories, is weaker than the claim that ontological naturalism is true, and remains a purely methodological thesis, there is a relatively straightforward argument *from* this consistent failure of supernatural theories in science, along with the success of their naturalistic rivals, *to* the conclusion that ontological naturalism is (probably) true (this inference is endorsed by Forrest, 2000 and Dilley, 2010).

The third view regarding MN recently defended in the literature is the unintelligibility view I have advocated (2020). I argue that supernatural claims and theories are unintelligible, not on traditional positivist grounds of unverifiability, but simply because we don’t have any clear idea of what it is to be natural (or supernatural). I consider various attempts to define ‘natural’ and ‘supernatural’ and find them wanting. The best hope, I suggest, is to link the natural to the physical. It is plausible that X is natural iff X is physical/material. (From now on I will follow this usage and refer to entities ruled out by MN as supernatural/immaterial (S/I) entities, and theories that posit them as supernaturalist/immaterialist (S/I) theories.) But it turns out, I argue, that we don’t in fact know what it is for something to be physical (or non-physical)—material or immaterial—either. I support this by reference to the well-known problem for physicalism known as ‘Hempel’s dilemma’ (Hempel, 1980): if the physical is tied to present physics, physicalism will very likely turn out false, given the probability of the theories and ontology of present-day physics being overturned in the future; but if it is tied to future, completed physics, it seems vacuous.^2^ I conclude that the categories of the ‘natural’ and the ‘supernatural/immaterial’ are hopelessly obscure, with the implication that explicitly S/I theories lack clear sense. This is not the case for supposedly ‘naturalistic’ theories however, since, I claim, standard scientific theories we may think of as naturalistic are not *explicitly* naturalistic theories, the way S/I theories are explicitly S/I theories. The former, unlike the latter, do not depend for their intelligibility on the categories of the natural and the supernatural/immaterial being meaningful. The upshot is that the entire debate around MN is misconceived, given that all forms of MN (and, for that matter, ontological naturalism), as well as their opposing views, are ill-defined, as they presuppose the intelligibility of the categories of natural and the supernatural/immaterial.

How does my view relate to IMN and PMN (aside from the obvious difference that IMN and PMN both regard supernatural claims as perfectly intelligible)? With IMN and against PMN, I hold: that there *is* something particularly problematic about the positing of S/I entities, that the S/I category is not just one metaphysically weird or challenging scientific category among others. There is no prospect, in principle, of the S/I category taking its place alongside indeterministic processes, wave/particle duality, or quantum entanglement, in the catalogue of legitimate and established scientific categories or phenomena, on my view. There is no prospect, in principle, of us discovering empirical evidence in support of S/I claims or theories.

With PMN and against IMN, I hold: that (despite what the above might seem to suggest) S/I claims or theories can be and have been part of science; that there is no defensible metaphysical demarcation criterion in terms of which to declare such theories inherently unscientific. They are pathological scientific theories, but they are or can be scientific theories nonetheless. With PMN and against IMN, I hold that S/I theories can and should be rejected directly, not just declared ‘unscientific’ in a way that leaves open that they might still be *true* (though for PMN they are to be rejected because the lack of evidence for the postulated supernatural entities or forces suggests they are false; for me the ill-defined nature of the category of the S/I means they should rather be rejected as unintelligible: not even false, we may say).

## Vitalism and methodological naturalism

### Vitalism as a test-case for methodological naturalism

‘Vitalism’ on the standard interpretation refers to a family of views in biology and natural history that has ancient roots, but which rose to prominence in the eighteenth and early nineteenth centuries, according to which the fundamental explanatory principle for life and living systems, the key element that distinguished organic from inorganic matter, was some kind of immaterial or non-physical substance or property, variously termed the vital fluid, vital force, vital spark, *elan vital*, vital principle, or ‘entelechy’, depending on which analogies to established physical entities, forces or processes were thought the most promising in characterising the immaterial component.^3^ Typically each species was thought to possess its own vital principle. Notable vitalists included Hans Driesch (whose early-twentieth century theory of entelechy is today probably the most widely-discussed and critiqued form of vitalism), Henri Bergson, Johannes Reinke, Johannes Peter Müller, and Oscar Hertwig.^4^

‘Vitalism’ is sometimes used quite broadly (for instance by Chen (2024) to refer to virtually any non-reductionist or non-mechanistic view regarding either the organic or inorganic realm, or indeed any view that doesn’t see matter (living or non-living) as passive or inert, but rather attributes some sort of active, or ‘vital’ principle or nature to it.^5^ This conflicts with standard usage on which vitalism is construed more narrowly as an explicitly S/I theory regarding specifically the living world. Although this is partly a terminological issue, I don’t regard the broader usage as particularly helpful. For my purposes, its most serious shortcoming is that it obscures the distinction between vitalism and *organicism*, a non-S/I anti-reductionist program in biology that I will discuss in more detail below. What Wolfe terms ‘functional vitalism’ (2011, 2017) or ‘non-metaphysical vitalism’ (2022) for example, is (as he himself recognises (2022, 2017), more or less indistinguishable from organicism^6^, as Chen also notes (2024, pp. 21–22).^7^ It will become clear later in the paper why I regard the differences between vitalism and organicism as important, and why the latter should not be subsumed as a variety of the former.^8^

Even on the narrower interpretation, recent scholarship has identified a great variety of vitalistic views and approaches in the history of the life sciences (see Chen, 2024, Ch. 1 for a summary of this literature). The differences between these various forms of vitalism are not very relevant for my purposes here. All that matters is that the extra ingredient required for life, that in virtue of which living things were alive, was thought to be immaterial, however else it was characterised. Vitalism in all its forms—at least as I shall understand it—was by its very nature an S/I theory.

Vitalism is rarely discussed in the literature on MN.^9^ The discussions are overwhelmingly preoccupied with religious or religiously motivated views such as IDC (and sometimes things like demonic possession).^10^ But vitalism would seem to be an ideal test-case for MN, for two reasons. First, however we understand MN, vitalism surely violates it. As I noted above, the entities or forces posited by vitalists were by their very nature S/I entities or forces. They may not have been divine or even ‘spiritual’ in the specifically religious sense, but these are not the only way to be supernatural or immaterial. Second, vitalism, not being, for the most part, a theory motivated by religious considerations, cannot be as easily dismissed as a potential piece of respectable science, compared to views such as IDC or demonic possession. Smith and Dawes (2018) argue that science has always eschewed supernatural causes or agency; but it is far from clear that the entities or forces postulated by vitalists count as ‘supernatural’ in their sense, thus their argument (which does not mention vitalism), even if correct vis a vis theories such as IDC, leaves the question of the status of vitalism untouched. Smith suggests elsewhere that the kind of entities prohibited by MN can be defined as ‘causally efficacious disembodied minds or immaterial agents such as ghosts, gods, demons, and hobgoblins’ (2017, p. 322; see Fales, 2013, who also defines the supernatural in terms of disembodied minds). But again, the entities posited by at least many typical versions of vitalism are not obviously covered by this definition (Boucher, 2020, p. 68). They are not minds. They may be immaterial *entities* or *forces*, but they are not typically personal or quasi-personal *agents* in the way that ‘ghosts, gods, demons, and hobgoblins’ are. Thus Haldane (1936, p. 38) notes that for vitalists, the vital principle operated ‘blindly’, making it quite different from older agential notions of ‘soul’.^11^ So either Smith would be happy to count such entities as natural and thus consistent with MN; or, if he would not (and I assume he would not; certainly he *should* not), his definition does not provide us with necessary conditions for an entity or force to violate MN. A theory of MN in the sciences that does not tell us whether and how to exclude vitalism is an inadequate one. (Of course, if I am right, the moral we should draw from these failed definitions of the supernatural/immaterial is not that we need a better account, but that no account is possible.)

In the following section I will consider vitalism in light of the three accounts of MN mentioned above.

### Vitalism, methodological naturalism, and the three views

According to IMN, vitalism, in postulating S/I entities or forces, was an inherently unscientific or pseudoscientific theory. It was not and could never be part of science.^12^ As a factual claim about the history of biology, this seems plainly false. Standard histories of biology include discussions of vitalism as a thriving and popular scientific theory during the eighteenth and nineteenth centuries, just as the theory of special creation must in any history of biology be recorded as one theory of the origin and character of species prior to Darwin, indeed one that continued to be accepted by some leading scientists even post the *Origin*. ‘Until about the middle of the nineteenth century vitalism continued to represent the usual belief among scientific men, including physicists and chemists who had given special attention to the phenomena of life’ (Haldane, 1936, p. 39). Indeed, as I intimated above, vitalism had an even stronger claim than creationism to be considered a part of legitimate science, since it was much less obviously influenced and motivated by religious convictions and traditions than was creationism. Of course, it is open for the defender of IMN to advance the normative claim that since vitalism was by its very nature an unscientific or pseudoscientific theory, it *should not have* been treated as a scientific theory, even if in fact it was. But since Kuhn (1996), philosophers of science have rightly been wary of advancing normative claims about the nature and process of science that seem to conflict with the actual history of science, and it is permissible to wonder if there is anything to be gained by retrospectively excluding vitalism from eighteenth and nineteenth century science, aside from saving IMN from refutation.

So far, this might seem to support the PMN interpretation of vitalism. According to PMN, vitalism was not and should not have been excluded from science due to its S/I metaphysics. The vital fluid or force was a potentially legitimate explanatory posit, evidence for the existence of which could, in principle, have been forthcoming.^13^ In fact no such evidence emerged, the theory ‘led to no advances during the course of [biologists’] researches’ (Hull, 1974, p. 128), rival theories were judged to be superior, and thus the theory was discarded, much the way the theory of phlogiston or the theory of caloric or the theory of the ether were discarded (McDonald & Tro, 2009). This failure then contributed to the poor track-record of S/I theories, a track-record that entitles us today to provisionally adopt MN. So the PMNer argues.

The problem with this interpretation (as IMNers would stress) is that the claim that there could in principle have been evidence for the existence of the vital fluid or force, much like there could have been evidence for the existence of phlogiston or caloric or the ether, is hard to take seriously. Unlike these other examples of failed scientific explanatory posits, and unlike non-pathological explanatory posits in general, S/I entities or forces have built into them, as it were, the impossibility of our obtaining empirical or experimental evidence of their existence.^14^ On the IMN view, this is expressed in terms of the claim that S/I claims or theories such as vitalism, while they are intelligible and might even, for all science knows, be *true*, are in principle scientifically untestable. On my view, such theories are not intelligible, and that is why they are (trivially) empirically untestable. They are not intelligible because the claim that an entity or force is immaterial or supernatural (or, for that matter, material or natural) has no clear sense.

My view also enables us to respond to a possible move the PMNer could make here. It is true that it is a central part of the standard argument for PMN that, *contra* IMN and my view, in principle we *could* discover empirical evidence for the truth of S/I theories or claims.

…one can easily imagine a collection of observations that would lend plausibility to the hypothesis of a supernatural origin of life … Suppose that species just popped into the fossil record without any discernible traces of evolutionary descent and without demonstrable relationship to other species… [or] that all available dating methods concurred on a 6000 year old earth and universe … . (Boudry et al., 2010, p. 241)

But someone sympathetic to PMN could argue that, at least when it comes to views such as vitalism, this is just the wrong way to think about it. In science it is rarely the case, at least in the first instance, that we have direct empirical or experimental evidence for the existence of postulated unobservable entities, forces or processes. Rather, these are postulated, at least initially, as *theoretical*-*explanatory* entities: their existence and behaviour would explain a range of observations, and that is why we should believe in them. And if the postulated entity comes to be rejected, as often as not it is because it is found to be *explanatorily unnecessary or redundant*, rather than (or at least, as well as) the fact that empirical or experimental evidence for its existence was not forthcoming. This was seemingly the case for epicycles, the ether, caloric, Aristotelian forms, and many others. And this seems the correct approach to assessing theories such as vitalism. While perhaps the discovery of lines from Genesis encoded into our DNA (Boudry *et al. ibid.*, 241) would constitute direct empirical evidence for IDC^15^, it is hard to imagine what an equivalent hypothetical item of direct empirical evidence in support of vitalism could be. But that, on the line of argument I am considering, is beside the point: the vital fluid or force was postulated as an unobservable, theoretical entity whose existence, properties and activity could explain the relevant observations about living systems, and it should be judged on that basis. Thus Carnap (1966, pp. 680-681) recalls that Driesch, in defence of his vitalist theory, claimed (Carnap is paraphrasing): ‘the physicist introduces forces that no one can observe—forces like magnetism and electricity—in order to explain certain phenomena. I wish to do the same’ (see Chen (2018, p. 4; 2024, p. 3) who draws a similar analogy to ‘immaterial’ forces and fields in physics). The problem with the posits of the vitalists, the defender of PMN might go on to say, was not primarily the lack of direct evidence for their existence, but the fact that they failed as *explanatory* constructs: they were deficient in the distinctively explanatory virtues. For instance, as has often been pointed out (e.g. by T.H. Huxley and Julian Huxley) there is a ‘dormative virtue’ quality to the postulation of an unobservable force or fluid to explain the phenomena of life about which all that can really be said is that it is what explains the phenomena of life. Carnap (1966) noted that the real problem with Driesch’s theory was that it provided no laws governing the behaviour of the entelechies it posited. It was thus explanatorily empty (unlike the theories of electricity and magnetism).

This then, is a more plausible way of developing the PMN view with respect to vitalism. On this view, the posits of the vitalists were rejected not, as IMN would have it, because they were metaphysically suspect (and thus intrinsically ‘unscientific’ or ‘pseudoscientific’), but because they were explanatorily inadequate.^16^ This is, perhaps, the sense in which they failed on standard scientific grounds, contributing to the woeful track record of S/I theories.

On my view however, the positing of S/I entities is unintelligible, so these questions of explanatory adequacy don’t even arise. The rejection of S/I theories on the ground that the S/I category is ill-defined is prior to the consideration of the specific strengths and weaknesses of the explanatory hypotheses involved. That is why there is a disanalogy between S/I posits and legitimate explanatory posits in science (of both the successful and unsuccessful kind), why, that is, the IMNers are right that there is something uniquely objectionable about S/I posits. As I put it:

[S]upernaturalist theories should be rejected not, as IMN would have it, because they violate naturalism understood as a meaningful metaphysical constraint, and not, as PMN would have it, because they fall down on the purely scientific grounds of empirical and explanatory adequacy, but because they are a priori unintelligible (so they can be rejected on grounds that are prior to consideration of their metaphysical or scientific adequacy). (2020, p. 68)

## The epistemic construal of the unintelligibility view, and its application to vitalism

### The epistemic construal of the unintelligibility view^17^

So far, I have presented the three views concerning MN, and considered what each would say about the case of vitalism. I have implied that my interpretation may be the most plausible one, but I do not claim that this case unambiguously establishes that it is superior to the other views. If one is firmly, independently committed to IMN, one is likely to be able to find a way to interpret vitalism that is consistent with it; the same goes for PMN. The case of vitalism is somewhat hard to square with these two accounts, I claim, but not impossible, if one is strongly committed to one of them on other grounds. In the remainder of the paper, however, I wish to consider vitalism in light of a somewhat different understanding of S/I theories, one that really does, I claim, have the potential to make clearer sense of what was wrong, and possibly what was right, in vitalism.

In the conclusion of my (2020) paper, I noted that the unintelligibility analysis presents a puzzle: ‘I have argued that supernaturalist theories are a priori unintelligible. Why then, are they proposed, debated, and, by some, believed?’ To answer this question, I proposed a somewhat different interpretation:

A supernaturalist theory or explanation is, I would suggest, in a sense not a theory or explanation at all, but a confession—perhaps a celebration—of ignorance. To invoke the supernatural is a way of saying: whatever our current scientific world picture is, whatever the set of explanatory principles or postulated entities, forces and processes of our best current science (or, presumably, any conceivable future science) may be, the phenomenon under consideration evades them. The supernatural is simply ‘something we know not what’… The mistake, then, has been to think of the supernatural as a metaphysical category, as denoting a particular ontological mode of being, when in fact it is an epistemic category: it is an expression of a lack of positive knowledge or understanding, and a pessimism about that knowledge ever being forthcoming; an admission of epistemic defeat, disguised (perhaps unconsciously) as a positive theory. (2020, p. 78)

These two interpretations we can call the *metaphysical* and the *epistemic* interpretations. On the former, a metaphysical claim is intended, but it misfires: an entity or force is posited which essentially belongs to a particular metaphysical category—the supernatural or immaterial—but since this category is hopelessly ill-defined, the positing of entities belonging to it is strictly unintelligible. On the second, epistemic interpretation, nothing is posited that belongs to a particular metaphysical category; rather, a purely epistemic claim is advanced. On this interpretation, nothing in the putatively S/I theory is unintelligible: certainly, something is posited that is claimed to *itself* be unintelligible, or at least unknowable, but there is nothing unintelligible about *this* claim.

It might seem like the metaphysical and epistemic interpretations are in conflict. I intended that they be compatible but did not say how the apparent conflict between them could be explained away. The natural way to do this is to distinguish a face-value and a non-face-value construal of the relevant claims. The face-value construal is the metaphysical one; the non-face-value construal is the epistemic one. To put it simply, when the vitalist says that life is explained by the existence of an S/I force or entity, what they *seem to* be saying, on face-value, is that life is explained by the existence of something that belongs to a particular metaphysical category. What they are, in some sense, *really* saying is that life is the result of factors or entities that are in-principle unknowable and undiscoverable by standard science.^18^

This kind of two-level picture, this distinction between a face-value construal and a construal that captures what is meant at a ‘deeper’ level, is after all not unheard of. When Chomsky suggested that ‘physical’ and ‘material’ are epistemic honorifics (the converse of my claim), such that to call something physical or material is in effect to say that it is understood or understandable, he was making essentially the same move (1994, p. 189). On his view, when someone claims that an entity, force or property is physical or material, they appear to be assigning it to a certain metaphysical category; but what they are really doing is advancing the epistemic claim that it is understood or understandable by us, or by standard science. When it is suggested (e.g. by Dilley, 2010) that to call something ‘scientific’ is to praise it, not to describe it, what is being claimed is that when someone calls a theory ‘scientific’ they appear to be assigning it to an epistemic category based on presumed objective features it possesses, but what they are actually doing is expressing a positive attitude towards it. For that matter, and similarly, emotivists and expressivists about ethics claim that when someone calls an action ‘good’ they appear to be ascribing a factual property to it, but what they are in fact doing is expressing a positive attitude towards it. Thus, the two-level picture is seemingly coherent and has philosophical precedents.

### The epistemic construal and vitalism

But why suppose the two-level picture of vitalism is *true*? Why ascribe this ‘deeper’ interpretation to the vitalist’s claims and commitments, when it seems to be so different from their claims and commitments on the face-value interpretation? In what follows I shall first present the move to the two-level picture of vitalism as one that makes sense given my unintelligibility view, but I trust that in the course of the discussion it will emerge that the view is independently plausible and has much to recommend it.

Supposing the argument of Boucher (2020) is correct, the metaphysical interpretation has the vitalist making strictly unintelligible claims. As I note, this makes it puzzling why such views would be advanced, debated, and by some, accepted. If we could find a way to reinterpret what they are saying as a claim that (1) it is reasonable to suppose they are in fact saying, (2) is intelligible, and (3) is not (or was not) obviously false, the principle of charity would seem to require us to do so.

The reinterpretation of the vitalist’s claim as the claim that life is the result of factors or entities that are in-principle unknowable and undiscoverable by standard science, arguably satisfies these three conditions. It is clearly intelligible. It was not *obviously* false at the time of its heyday. It, to be sure, came to be regarded as false by most biologists by the late nineteenth and early twentieth centuries. On the interpretation offered here, its opponents rejected vitalism not primarily because it invoked a certain problematic metaphysical category, but because its implicit claim that life is the result of factors or entities that are in-principle unknowable and undiscoverable by standard science, was overly pessimistic (since, of course, it is not just the vitalists, but their opponents, who were engaged in debating this implicit epistemic claim, on the meta-thesis in question). And this assessment is accepted today. But when it was in the ascendency, it was endorsed by many respectable scientists and philosophers, and this was not unreasonable given the evidence at the time.^19^ Indeed we shall see that from the organicist point of view, there is a true claim in the vicinity of the strongly pessimistic vitalist claim that is less pessimistic: that life cannot and never will be fully explained using *mechanistic* science. So the epistemic reinterpretation satisfies (2) and (3).

The argument for it satisfying (1) is that when we look at at least some of the actual debates from the time, it is manifest that what is in fact being debated is not vitalism understood as a metaphysical claim, but as the epistemic claim. Thus Nicholson and Gawne note that ‘Haldane had pointed out that the vital force was simply ‘a convenient resting-place’ for the facts about the organism that mechanical explanations could not explain’ (Nicholson & Gawne, 2015, p. 358. Note the reference to the inadequacy of *mechanical* explanations; I shall return to this.) It often seemed that it was the fact that entities or forces postulated by the vitalists were ‘unknown’ or ‘unknowable’, rather than *metaphysically suspect*, that constituted the most serious mark against them. So we find that Ritter describes vitalism as a ‘walled city with the gates locked and the keys lost beyond recovery’ (Ritter, quoted in Nicholson & Gawne, 2015, p. 359). On these construals vitalism is not so much advancing a positive claim to the effect that life *could be* explained by invoking nonphysical forces or entities, but rather the negative claim that life *could not* be explained by current (or future) science, or, a weaker claim that we shall consider presently, by mechanistic science. Haldane notes that vitalism ‘only amounted to a negative protest against … mechanistic physiology…’ (1936, p. 41).

It doesn’t matter, for the sake of *this* argument, that this was not the case across the board. I obviously can’t and don’t deny that much of the debate appeared to concern the propriety and fruitfulness of positing entities belonging to a particular positive metaphysical category. All that we require to support (1) is that *sometimes* the epistemic claim seemed to be what was in question. Indeed given the two-level picture, we would *expect* the debate to at times concern vitalism construed in face-value metaphysical terms, and at others, as construed in epistemic terms.

Thus (1), (2) and (3) all appear to be satisfied. I conclude vitalism can reasonably be construed in epistemic terms.

## Vitalism as mysterianism

On the epistemic interpretation of vitalism, the view amounts to the claim that there is something that explains life, but it cannot be, and will never be, understood by standard science. The positive metaphysical characterisation of this force or entity—as immaterial or supernatural—drops out, and all that is being advanced is this negative epistemic claim. On this interpretation the view is similar to so-called ‘mysterianism’ about consciousness, the view that consciousness will always remain a mystery to us. The difference of course is that mysterianism about consciousness is an *explicitly* epistemic-pessimistic view, while vitalism *appears to be* a positive metaphysical-scientific theory, and is only revealed as an epistemic view upon analysis and reinterpretation, such as I have given.

Of course, on the metaphysical interpretation of vitalism, the obvious analogy for the philosophy of mind is dualism, not mysterianism, and vitalism has often been associated with forms of dualism about mind. But we are working here with the epistemic interpretation of vitalism, for which mysterianism, not dualism, is the appropriate analogy. Indeed if the epistemic interpretation is an acceptable construal of vitalism, it is reasonable to suppose that a similar reinterpretation of dualism in the philosophy of mind as mysterianism-in-disguise may be defensible (given that the invocation of non-physical substance or properties here is, on my view, just as unintelligible as in the case of vitalism; see Boucher, 2020, p. 69), but I will not pursue that thought here.^20^

Mysterianism about consciousness comes in a strong and a weak form. According to the strong form, consciousness will always remain a mystery to us. Our ignorance of consciousness is, as Stoljar (2006) expresses the view, ‘chronic’. The most prominent defender of strong mysterianism is McGinn (1989, 1991). McGinn argues that we are ‘cognitively closed’ with respect to consciousness. We are simply not equipped with the cognitive ability to solve the philosophical problem of consciousness, just as dogs lack the ability to understand advanced mathematics: ‘We are cognitively closed to certain truths, not because they are inherently unknowable, but because of the kind of mind we possess’ (1991, p. 2). It is important to note however that this claim about cognitive closure is a further claim, distinct from the bare claim that consciousness will always remain a mystery to us. It is an explanation for this latter claim, but one could accept that consciousness will always remain a mystery to us (perhaps as a straightforward induction from our failure to make any progress in solving the problem) without accepting this further claim about cognitive closure. It may of course be that without supposing something like cognitive closure, the claim that consciousness will always remain a mystery lacks motivation or plausibility. It’s not clear *why* it should forever remain a mystery unless something like cognitive closure is true. But they are still distinct claims.

There is a weaker version of mysterianism however, one defended by Stoljar (2006). On this view we are *currently* ignorant of some non-experiential fact or truth that is relevant to experience (consciousness), but there is no reason to think this fact must be in principle undiscoverable by us, and thus no reason to suppose our ignorance must be chronic.^21^ Consciousness need not forever remain a mystery (hence he prefers the name ‘ignorance’ or ‘epistemic’ theory rather than ‘mysterianism’), though of course, it might—this can’t be ruled out. But we need not posit cognitive closure in McGinn’s sense. Pessimism of this sort is, he claims, unwarranted.

The way we have been discussing the epistemic interpretation of vitalism, it is clearly akin to the strong version of mysterianism: the claim that life will forever remain a mystery, that it will never be explained by standard science. To express it in Stoljar’s terms, we are ignorant of the correct explanation of life, and chronically so. One *could* add the further claim about cognitive closure: that we are cognitively closed with respect to the basis of life. But as I noted above, this would be a further claim, not logically required by mysterianism as such (and, needless to say, it was not explicitly articulated by vitalists given that, unlike mysterianism about consciousness, vitalism tended not to be expressed in explicitly mysterianist terms).

## Vitalism, ignorance, and organicism

### A weaker ignorance claim?

The upshot, it seems, is this: vitalism understood as strong mysterianism about life is intelligible, but false. We may have been ignorant about the basis of life at one point, but we are no longer. The claim that we are chronically ignorant of this, while perhaps understandable at an earlier period (Chalmers argues that views such as this were quite ‘natural’ at a time when ‘very little was known about the enormous sophistication of biochemical mechanisms’ (1996, p. 109)) turned out to be premature, and overly pessimistic.

We need however to consider an alternative way of construing vitalism on the epistemic interpretation, according to which it is more similar to Stoljar’s weaker ignorance hypothesis regarding consciousness. It is clear that vitalism was, fundamentally, a critique of *mechanistic*-*reductionist* approaches to life (Haldane, 1936, p. 38). What the vitalists wanted to defend above all was the autonomy of biology, the idea that life and biological phenomena needed to be explained on their own terms, on the basis of irreducibly biological laws and principles, that they could not be reduced to or explained entirely in terms of physics or chemistry. They are on the holist side of the perennial holism vs. reductionism divide in the history of biology (Gilbert & Sarkar, 2000, p. 1; Hein, 1969). As Carnap notes, for Driesch, the vital force ‘is not spatially located. True, it acts on a physical organism, but it acts *in respect to the entire organism*, not just to certain parts of it’ (1966, p. 681; italics added).

This suggests a modified version of vitalism understood in epistemic terms: the claim was that life—‘particularly the manner in which organisms maintain, heal, and reproduce themselves’ (Haldane, 1936, p. 38)—would not and could not be explained by *reductionist*, *mechanistic* science. Now this could still come in a strong-mysterian form: the vitalist could claim that our ignorance concerning the non-reductionist, non-mechanistic facts that would account for life is chronic. But it could equally suggest a characterisation of vitalism as something closer to the Stoljerian-consciousness view: the view that we are *currently* ignorant of the basis of life, but that this ignorance need not be irremediable. Our ignorance will not and cannot be remedied, however, by reductionist, mechanistic science. Put in Stoljar’s terms, the claim is that there is some non-mechanistic truth or set of truths that is relevant to explaining life, of which we are presently ignorant, but that we could and perhaps will come to know.

According to those who defended organicism—what we can think of as the successor holist program to vitalism in biology—the claim that life could not be fully explained in reductionist or mechanistic terms (in terms of physics and chemistry) was entirely justified. Organicism was an anti-reductionist research program in biology and the philosophy of the life sciences that flourished especially in the early part of the twentieth Century, and was associated with thinkers such as E.S. Russell, J. S. Haldane, J.H. Woodger, W.E. Ritter and L. von Bertalanfy (see Nicholson and Gawne (2015) for a discussion of their views). According to organicism, there is indeed a principle necessary to fully explain life, that is not a mechanistic principle, and does not derive from physics or chemistry: namely, the principle of *organisation*. Our ignorance of the basis of life can be remedied, but only by way of this principle, organicists argue. Thus to the extent that the vitalists were insisting that there was some non-mechanistic truth relevant to explaining life, they were correct (recall again the Haldane quote about the vital force being a ‘convenient resting place’ for these non-mechanistic facts); but the claim that we are ignorant of this truth, at least once organicism is on the table, is incorrect: we are in possession of it to the extent that we grasp the (non-S/I) principle of organisation. Organicism (according to its advocates) takes from vitalism the important insight that mechanistic science is inadequate to capture the nature and complexity of life and living systems; but goes beyond vitalism (on the epistemic construal) in positively supplying the missing ingredient, in its appeal to the (non-S/I) notion of organisation.^22^ For now we can think of this as the idea that it is the way living systems (with organisms as the paradigms) are organised, as integrated, cohesive, adaptive, purposive unities or agents, with emergent properties and behaviour (they are more than the sum of their parts), typically exhibiting functional differentiation and division of labour, that is the key to understanding them *qua* living systems, rather than the mechanistic-reductionist approach that sees them as ‘mere aggregates of mechanical parts’ (Walsh, 2015, p. 41). Organisation also involves, as Mossio puts it, ‘a generative dimension in the form of a mutual dependence, such that the very activity and existence of each organized part depends on its mutual relationship with the others’ (Mossio, 2024, Introduction, p. 10).

This principle of organisation involves more than the recognition that living (and perhaps some non-living) things are organised, highly intricate and complex.^23^ This last claim is, of course, accepted by mechanistic reductionists. The difference is that for the latter, this organised character of systems is something to be explained, using reductionist methods, i.e. complex wholes are to be understood and explained in terms of the properties, relations and interactions of their parts. For organicists, organisation is to be invoked as *explanans*, not just treated as *explananda*. It is itself a crucial, independent and indispensable theoretical principle that can help to explain the character and behaviour of especially living systems. And, as noted above, it is a holistic, non-reductionist principle, permitting top-down, emergentist and holistic forms of explanation. As Nicholson puts it, with respect to organisms: ‘The properties and behaviour of the parts are partially determined by the organization and activity of the organism as a whole’ (Nicholson, 2014, p. 353). This top-down, holistic understanding of organisation and its explanatory role is not consistent with mechanistic reductionism, which is necessarily a bottom-up approach.

### Non-life facts and the autonomy of biology

There might seem to be an important disanalogy between mysterianism or the ignorance theory of consciousness on the one hand, and vitalism construed in epistemic terms on the other. The way Stoljar expresses it, the former is the view that there is some *non-experiential* truth relevant to experience, of which we are ignorant, either chronically or temporarily. This suggests that vitalism construed in epistemic terms ought to be the view that there is some *non-life* (or non-biological) fact relevant to explaining life, of which we are ignorant, either chronically or temporarily. But doesn’t this contradict the vitalist commitment to the autonomy of biology, alluded to above, according to which biological phenomena need to be explained on their own terms, on the basis of irreducibly biological laws and principles? And isn’t the positive proposal of the organicists—that the principle of organisation is the missing ingredient—a principle of living systems only? I think this is a mistake. To secure the autonomy of biology it is not necessary that the principles, laws, processes etc. invoked be unique to the living world. It is sufficient that they not be mechanistic-reductionist, physico-chemical laws, principles, processes etc. The principle of organisation applies, so far as we know, principally and paradigmatically to living systems. But it is not itself a purely biological principle, it is more abstract and general than that. It is in-principle applicable to the non-living world, even though, as far as we know, it is primarily living systems that are as a matter of fact organised in this sense. This is shown by the fact that, as Mossio points out (2024, Introduction), the principle of organisation has been invoked by contemporary organicists to help explain the origin of life. He notes that this ‘metabolism-first’ approach ‘does presuppose organization as an *explanans*, under the general hypothesis that some degree of organized complexity is actually required to boot-strap an evolutionary process leading to the appearance of living systems as we know them’ (ibid., 8). It follows that as a principle, organisation is more general than, and does not presuppose, or only encompass, life and living systems.^24^ Thus, I take it that the principle of organisation is, strictly speaking, a ‘non-life’ or ‘non-biological’ truth relevant to explaining life and living systems, as required by the views in question.

### Defusing modal intuitions

Central to Stoljar’s defence of the ignorance view with respect to consciousness is its ability to defuse modal arguments for forms of dualism or the non-supervenience of experience on the non-experiential (which is often thought of in terms of the ‘physical’ but need not be) (2006). This is because the problem of consciousness that the epistemic view is designed to solve, is the problem of reconciling the plausibility of such modal arguments with the plausibility of arguments for the supervenience of the experiential on the non-experiential. On the ignorance view, modal arguments for non-supervenience seem compelling, but are mistaken. These arguments suggest that we can imagine all the non-experiential facts obtaining without the experiential facts obtaining (for example ‘conceivability arguments’ such as Chalmers’ famous zombie argument, 1996), thus showing that the experiential facts are something over and above the non-experiential facts, some extra ingredient of reality. The mistake in these kind of arguments, according to the ignorance view, is the supposition that we are now in a position to imagine all the non-experiential facts. If there is a set of non-experiential truths relevant to experience of which we are ignorant, then these truths are necessarily excluded when we attempt to imagine all the non-experiential facts. We may be able to imagine that the non-experiential facts *minus* these facts of which we are ignorant might obtain without the experiential facts obtaining. But this does not demonstrate that we could imagine the non-experiential facts *including* the facts of which are currently ignorant obtaining without the experiential facts obtaining. It thus does not establish failure of supervenience, on the ignorance view.

This can be applied to vitalism and organicism in the following way. Take the full set of mechanistic and physico-chemical facts relevant to living systems. It might seem that we can imagine these facts obtaining without anything being alive. This is the modal intuition.^25^ The mistake here, according to vitalism on the mysterian or ignorance interpretation, is in supposing that in imagining all the mechanistic and physico-chemical facts, we have imagined all the non-life facts relevant to living systems. It is plausible that there is a set of non-mechanistic, non-physico-chemical truths relevant to life of which we are ignorant and which, in combination with the mechanistic and physico-chemical facts, suffice to explain life. We need not think that it is conceivable that this larger set of facts might obtain without anything being alive.

The strong mysterian version of vitalism goes on to suggest that our ignorance of these further facts is chronic. On the weaker Stoljerian version, we are presently ignorant of them, but our ignorance may be remediable. And according to organicism, it has in fact been remedied: the (non-S/I) facts concerning organisation are what we need to add to the mechanistic and physico-chemical facts in order to fully account for the nature and behaviour of living systems. As Russell, an early twentieth century organicist, noted:

[W]hile many of the phenomena presented by living things are […] to be explained as the direct result of simple physical and chemical relations, there still remain a vast number of facts of life which cannot be explained by any direct reference to chemical laws. (Russell, 1911, p. 332)

It is important to understand that this is a metaphysical claim about reality, not just an epistemic claim about the limitations of our knowledge, or the best way for *us* to construct explanations.^26^ The organicists are not just claiming that in practice we have no choice but to advert to non-physico-chemical principles, properties and processes, such as the principle of organisation, in order to explain life, given the practical impossibility of deriving all the facts about living systems from physics and chemistry. They are making the stronger claim that there are, in the world, real, objective, higher-level facts about living systems (such as those pertaining to their organisation) that cannot, in principle, be captured in physico-chemical terms. Whether this amounts to a denial of the supervenience of the biological on the physical, broadly understood, depends on what is meant by ‘the physical’. I have argued (Boucher, 2020) that there is good reason to think that talk of the physical in general doesn’t mean anything at all, as I noted above, so I regard this question of the supervenience or otherwise of certain things on the *physical* as a pseudo-question. But I set this question aside for now.

### Vitalism: what it was, and what it should have been

To return to our interpretive question about vitalism, whether the vitalists tended to be strong mysterians, or were only committed to the weaker Stoljerian claim that there is some non-mechanistic, non-life truth relevant to explaining life of which we are *currently* ignorant (or whether some accepted the former and some the latter), is a question on which I shall remain agnostic. Sometimes vitalism is interpreted as the view that the principles that would explain life are unknowable, which suggests the strong reading. Thus Nicholson and Gawne note that from the standpoint of organicism, ‘The vitalists had been right to defend the autonomy of biological theory, but they had been wrong to ground it in the supposition that the characteristic features of organisms are derived from the activities of *unknowable* directive agencies’ (2015, p. 358; emphasis added), while Mayr suggests that vitalism ‘fall[s] back on an unknown and *presumably unknowable* factor…’ (Mayr 1982, quoted in Chen, 2024, p. 8; emphasis added). On the other hand, at least some varieties of vitalism seem to be consistent with the claim that non-mechanistic principles on the basis of which life could be explained may one day be forthcoming.^27^ For instance, Wuketits suggests that what he calls the ‘naturalistic’ vitalists ‘have not regarded the ‘life force’ as a mystic and unintelligible factor’; rather, they advocate searching for ‘organic laws transgressing the range of physical explanations’ (Wuketits, 1989, quoted in Chen, 2024, p. 19).^28^

As I noted above, from the standpoint of organicism, the claim that there is some non-mechanistic, non-reductionist, non-life truth relevant to explaining life of which people are *currently* ignorant (the weak ignorance thesis), was correct so long as the principle of organisation was unknown or unappreciated, but false once it came to be known and appreciated. What if vitalism is interpreted rather as committed to the strong form of mysterianism? In that case, according to organicists, they went too far. Such pessimism was premature. The more limited ignorance claim expresses what, according to the organicists, the vitalists, in a sense, *should have* said: the most that they were *entitled* to believe. It captures what was *right* in vitalism.

One question here is *when* the principle of organisation and its significance came to be understood. Versions of the (non-S/I) principle of organisation can, it is true, be found in the work of earlier philosophers such as Aristotle, and especially Kant, in his *Critique of Judgement*:

[O]ne wheel in the watch does not produce the other, and even less does one watch produce another, using for that purpose other matter (organizing it); hence it also cannot by itself replace parts that have been taken from it, or make good defects in its original construction by the addition of other parts, or somehow repair itself when it has fallen into disorder: all of which, by contrast, we can expect from organized nature … [A]n organized being is thus not a mere machine, for that has only a *motive* force, while the organized being possesses in itself a *formative* force (*Bildungskraft*), and indeed one that it communicates to the matter, which does not have it (it organizes the latter): thus it has a self-propagating formative power, which cannot be explained through the capacity for movement alone (that is, mechanism). (Kant, 1790/1987: § 65; quoted in Mossio, 2024, p. 12)

Thus it might be that, in some sense, the principle of organisation, shorn of any explicit S/I content, was available at the time vitalism was in the ascendency.^29^ But it is not, I’d suggest, inaccurate to say that until it was taken up, articulated and defended by organicists, biology in general (perhaps science in general) was largely ignorant of the nature and significance of the principle for the science of life.^30^

### The ‘not scientifically useful’ objection

It is against this background that we can understand one of the most common objections to vitalism, the practical or methodological one that it was not scientifically useful or fruitful, that it didn’t take biology forward, that it blocked empirical and theoretical progress, that it didn’t provide a workable research program that could be put into practice. Thus Nagel writes (1951, p. 327): ‘Vitalism of the substantival type… is now a dead issue… less, perhaps, because of the … philosophical criticism that has been leveled against the doctrine than because of the infertility of vitalism as a guide in biological research and because of the superior heuristic value of alternative approaches.’ This will be true of all strong mysterian views, of course. Even if mysterianism about consciousness is *true*, as a philosophical claim, it obviously does not (and does not aim to) specify a workable program of research for the sciences of the mind: indeed, what the view asserts is precisely that there can be no successful scientific theory of consciousness (at least for humans). Interpreting vitalism as strong mysterianism thus makes it especially clear why this objection—that vitalism is not scientifically fruitful—was so widespread and convincing.

But what about the weaker, ignorance interpretation? From the standpoint of organicism, while the claim that there is some non-life, non-mechanistic, non-physico-chemical fact of which we are currently ignorant (but which is potentially discoverable), which is relevant to explaining living systems, may not *in itself* have taken biology forward, it was or would have been potentially scientifically useful in posing the right questions for researchers, and pointing them in the right direction (or at least away from the wrong direction). While it didn’t itself provide the positive alternative, it was or would have been valuable in highlighting the limitations of the reductionist-mechanistic approach to life, and thus helping to clear the ground for a future, more adequate, non-mechanistic biology.^31^ This challenge was then taken up by organicism.^32^ So, again, from the perspective of organicism, if vitalism is understood as committed only to the more modest ignorance view, the objection that it was an impediment to progress in biology, would have lost much of its force.

### Vitalism and contemporary organicism

Is this only relevant to the historical question of the relationship between the two mistaken and superseded biological programs of vitalism and organicism? Were not the organicists just as mistaken as the vitalists in supposing that life could not be explained in mechanistic, physico-chemical terms? The standard, textbook story about the demise of vitalism and the rise of modern biology and genetics is that it was precisely the increasing and undeniable success, in the twentieth century, of the project of explaining life in entirely physico-chemical terms (with the discovery of DNA and the molecular revolution, of course, being the culmination of this) that doomed the vitalist project. On this view, the claim that life cannot be explained by reductionist, mechanistic science—that is, that there is some non-mechanistic, non-physico-chemical truth, currently unknown, that is relevant to explaining life—was never correct. If, of course, the claim was merely that there was some non-life truth or set of truths, currently unknown, that was relevant to explaining life, it would have been correct; at least, until the relevant physico-chemical truths came to be known. But nobody supposes that vitalists were only advancing *this* claim.^33^

But it is not of only historical interest. Organicism has in recent years enjoyed a significant revival in biology and the philosophy of biology (Walsh, 2015, 2018; Nicholson, 2013, 2014; Nicholson & Gawne, 2015; Nicholson & Dupré, 2018; Dupré, 2021; Huneman, 2010; Reiss & Ruse, 2023; Mossio, 2024; Mossio & Moreno, 2010; Mossio & Bich, 2017; Moreno & Mossio, 2015; Gilbert & Sarkar, 2000, Laubichler, 2000). The new organicists urge a move away from the gene-centrism of orthodox modern synthesis neo-Darwinism and the reductionism of molecular genetics, and in the spirit of their organicist forebears, argue for a re-centreing of the organism, understood in a holistic sense, and more generally the idea of organisation (as discussed above), in evolutionary theory and other areas of the life sciences. They argue that life and living systems are more than physics and chemistry: biology is an autonomous science. Organisms are, on this view, more than collections of molecules, or even assemblages of cells. Evolution is more than the adventures of genes.

I am sympathetic to the new organicism, but cannot defend it here. *If* the new organicists are right, the vitalists were indeed correct in supposing that life could not be explained in reductionist, mechanistic terms, and the earlier organicists were also right in thinking that the principle of organisation was the key non-mechanistic explanatory principle in biology. But there have been important developments in organicist thought. The new organicists are not just repeating what their predecessors in the early twentieth century were saying. Take the principle of organisation. Even in the earlier period, there were concerns among some organicists that the principle, as it was often appealed to, was too vague, too unspecified or unanalysed, to take us much beyond the empty and unhelpful posits of the vitalists (Nicholson & Gawne, 2015, pp. 365-366). Organisation couldn’t *just* be *explanans*; it also needed to be treated as *explananda* (ibid.). Among new organicists, this problem has been posed more sharply, and attempts have been made to address it. For instance, in the introduction to the recent collection *Biological Organisation*, Mossio notes that while the notion of organisation is promising and suggestive, and is increasingly being appealed to by theorists pursuing anti-reductionist agendas in a number of areas of biological science, when it comes to coherent articulation and illuminating analysis it remains something of a ‘blind spot’, even for influential programs such as the so-called ‘Extended Evolutionary Synthesis’, with which it is most commonly associated (Mossio, 2024 , p. 6). Mossio’s own proposal is to explicate the notion of organisation in terms of the notion of *autonomy* (Moreno & Mossio, 2015). Other theorists sympathetic to organicism, such as Dupré (2021) have been more sceptical of the notion of autonomy, preferring to think of organisms as *processes* (rather than autonomous substances or things) embedded in larger processes, where the boundaries between these processes are fuzzy and context-relative.

There is much more we could say about recent work within the organicist tradition, but the key point here is just that organicism is a lively and respectable program in contemporary biology and philosophy of biology, from the point of view of which a reassessment of the virtues (and failings) of vitalism, as a partly correct fellow anti-reductionist and anti-mechanist program, appears to be called for (Chen, 2024; Walsh, 2018). The epistemic interpretation of vitalism, I have argued, allows us to do this.

## Vitalism, intelligent design creationism, and mysterianism

A concern may be raised at this point that if the above account of vitalism is correct, it may license a similar reinterpretation and partial rehabilitation of IDC, something I certainly would not want to countenance. If some S/I theories were in fact partially correct, will that overgeneralise to S/I theories less deserving of our generosity?

But the arguments do not transfer to IDC. If we were to reinterpret IDC in epistemic, or mysterian, terms, it would be limited to the claim that there is some fact or set of facts, not captured by any version of contemporary evolutionary theory, relevant to explaining, say, the traits of organisms, of which we are ignorant, either temporarily or chronically. It is open to us simply to deny that this is the case. But more to the point, this is an implausible construal of IDC. IDCers do not think we (or at least they) are ignorant of the relevant facts: they are known, broadly, as intelligent design facts. IDC *tells us* what evolutionary theory leaves out: the truths about intelligent design (even if the intentions, modes of action etc. of this designer are left vague). It is thus more closely analogous to organicism, in attempting to supply the ‘missing’ ingredient required to complete our understanding of the living world and its history. And the appropriate response, of course, is simply to either deny that there is any such missing ingredient, anything contemporary evolutionary theory leaves out, or claim that even if there is, intelligent design is certainly not well-equipped to supply that deficiency. If it be objected that vitalism also tells us what mechanistic science leaves out—the vital force or fluid—I would reply that to the extent that all it tells us about it is that it is an S/I force or entity that explains why living things are alive, it hasn’t succeeded in positively characterising it in intelligible terms. Hence the need and justification for the epistemic reinterpretation. The notion of intelligent design, on the other hand, is perfectly intelligible. Of course, on my view, the claim that the designer and her actions are supernatural or immaterial, is unintelligible. But while this is, of course, what IDCers in fact tend to believe, IDC can, in principle, be formulated in such a way that all it is committed to is the idea of intelligent design itself, not to any claims about the S/I character of the designer or her activities. Perhaps the intelligent designer is a super-intelligent alien, rather than a divine or supernatural being, for instance (Boucher, 2020; Sober, 2007).

## Conclusion

Vitalism in biology is an under-explored test-case for views concerning MN. In this paper I have suggested that interpreting vitalism in terms of the epistemic version of the unintelligibility view is illuminating both with respect to understanding vitalism itself, and in terms of grasping its relationship to organicism: something that is of significant contemporary interest given the recent flourishing of organicist programs in the life sciences and their philosophy. This provides some support for the general validity of my view. But this interpretation of vitalism is also valuable as an antidote to still all-too-prevalent whiggish accounts of the history of biology on which the vitalists were deluded, muddle-headed mystics, with nothing of intellectual value to contribute: vitalism as a regrettable episode at the infancy of modern biology that is best forgotten (Chen, 2024, pp. 4–9; Wolfe, 2022, 2017). Even Walsh, who in a recent paper defends a methodological version of vitalism (see footnote 30), agrees that in its non-methodological form vitalism was ‘daft’ (2018, 167). It deserves more credit. Interpreted as a version of strong mysterianism—especially as the view that we are chronically ignorant of the *non-mechanistic* facts that would explain living systems—vitalism emerges as a coherent and reasonable conjecture at that juncture of history, albeit one that in hindsight we can recognise was overly pessimistic, and that, as the critics correctly pointed out, could be of little service to actual research in biology. If interpreted rather as a Stoljerian ignorance claim, vitalism emerges as a justified, arguably correct (at one time), and most importantly, scientifically fruitful claim, one that correctly identified limitations to mechanistic-reductionist approaches to life and living systems, and posed the right questions for future research, questions to which subsequent anti-mechanist programs in biology such as organicism, many now believe, have provided convincing or at least promising answers.

## Data Availability

Not applicable.

## Footnotes

1 As it is sometimes expressed, on this view IDC is unwelcome in contemporary science not because it is non-science, or pseudoscience, but because it is *dead* (discredited) science.

2 I also consider and reject what I call the ‘paradigm-case’ approach to defining the physical, which suggests we can pin down our understanding of the physical by tying it to some intuitively clear ‘paradigm’ cases of physical, and non-physical, entities. See Donahue (2024) for a defence of the paradigm-case approach against my critique.

3 As with contemporary dualism about consciousness, historically one of the main arguments offered against vitalism (for instance by members of the Vienna Circle) appealed to the causal closure of the physical (Wolfe, 2022, p. 3). This argument assumes we have a clear grasp of what ‘the physical’ is, something that I deny (2020).

4 For recent discussions of vitalism see Chen (2024, 2018); Wolfe (2011, 2017, 2022); Donahue and Wolfe (2023), and Normandin and Wolfe (2013).

5 This has given rise to a debate about the extent to which Aristotle (among others) may have been a vitalist (Wolfe, 2022, p. 2, Chen, 2024, p. 9).

6 Wolfe (2022) restricts the term ‘vitalism’ to views about the distinctive features of life (unlike some authors), but does not restrict it to S/I views.

7 Chen’s complaint about this is that distinguishing ‘functional’ from ‘substantival’ (S/I) vitalism permits Wolfe to unjustifiably accept the former and reject the latter (Chen, 2024, pp. 21 − 22).

8 Organicists themselves have thought it important to sharply distinguish their views from vitalism, but we shall see that the way they tend to draw the distinction is quite different from the way I propose to.

9 Exceptions include McDonald and Tro (2009) and Donahue (2024).

10 Thus one of the important contributions to the recent literature on MN, Boudry et al. (2010), has IDC in the title. The five examples Donahue (2024) gives of claims that may violate MN are all religiously motivated posits, involving gods, demons, ancestor-spirits, and souls leaving bodies. Later in the paper I will suggest that IDC can be formulated in a way that does not commit it to any supernatural, and thus religious, claims. But to the extent that it does invoke the supernatural, what is being invoked is, of course, a divine creator-god.

11 See Benton (1974) for a taxonomy of forms of nineteenth century vitalism that classifies them in terms of how they understood the teleological, rational, agential, and nomological character of the vital forces or entities they posited.

12 Wolfe (2022, p. 6) mentions the ‘frequent claim, visible in Bichat, Bernard and others, that vitalism had good intentions but in the end was just not a scientific theory, but a metaphysics of life.’

13 At least, this is how one would expect the PMNer to argue, given what PMNers have said in this past. Below I will consider a different and possibly more defensible approach PMNers could take.

14 It is sometimes claimed that there *were* experimental tests devised for vitalism, but on closer inspection what was really being tested, I would suggest, was the sufficiency or otherwise of mechanistic, physico-chemical processes, forces or laws to account for living systems, not the existence or otherwise of vital entities *qua* S/I entities (Chen, 2018, p. 5). The sufficiency of mechanistic, physico-chemical processes, forces or laws to account for living systems (even if that could somehow be experimentally proven) is neither necessary nor sufficient for the rejection of vitalism. It is clearly not necessary since its denial is consistent with organicism, as we shall see. More surprisingly, it is not sufficient either, since one could, in principle, hold that mechanistic, physico-chemical processes, forces or laws are sufficient to account for living systems, while still believing in vital entities, much like one could hold that physical events entirely suffice to cause and explain human behaviour while also holding that there are, in addition, non-physical mental events or properties (e.g. epiphenomenalism). Certainly in such a case vitalism would lose much of its point and motivation. But note that this very idea of empirically testing vitalism presupposes that the concept of vital, S/I entities has coherent sense, something I deny.

15 In fact even this is questionable; see Boucher (2020). But I am accepting it for the sake of argument.

16 Thus Chen (2018), though not defending PMN, argues that vitalism was not considered a ‘metaphysical heresy’ for positing S/I entities or forces, but was rather rejected on the scientific grounds (he somewhat confusingly calls them ‘logical’ grounds) of its inability to specify vital laws (as in Carnap’s objection mentioned above), or experimentally demonstrate the inadequacy of deterministic mechanistic explanations.

17 For convenience I shall refer to this as the epistemic construal of the unintelligibility view, though we shall see that on this construal the relevant claims are not in fact unintelligible

18 Below I will consider whether vitalism can be understood as committed to the weaker claim that while the explanation for life is currently *unknown*, it need not be *unknowable*: we may come to know it.

19 Note also that some of the standard objections to vitalism that we have looked at simply do not apply on the epistemic construal. For instance, the old ‘dormative virtue’ objection: on the epistemic construal, the vitalist is not positing an entity or force about which all that can be said is that it is the explanation of life. Nothing specific is being posited at all. Rather than vitalism offering poor or empty explanations, it is claiming that for us, there *is no* or *can be no* explanation.

20 The claims, firstly, that dualism about consciousness is really a form of, or closely related to, mysterianism and secondly, that dualism/mysterianism about consciousness is akin to vitalism, have been made by others (Dennett (2006, pp. 8–9) groups mysterians and dualists together under the heading ‘reactionaries’, and in his (1991, p. 281) he connects these views with vitalism; see Chen, 2024, p. 7; Wolfe, 2022, p. 4). The purpose of the comparison, in this case, is, of course, to tar dualism/mysterianism about consciousness with the brush of vitalism: just as the latter turned out, as we all now recognise, to be premature and overly pessimistic regarding our ability to scientifically explain life, we should also reject the former as being overly pessimistic regarding our ability to scientifically explain consciousness. But this ‘companions in guilt’ line of argument is not necessitated by the comparison. It is perfectly possible and defensible to argue that mysterianism about consciousness is more plausible than mysterianism about the basis of life. With respect to consciousness, it is the first-personal, subjective, phenomenal, ‘what it’s like’ character of experience that makes it uniquely challenging to fit into our current scientific world-picture, it may be argued. This was never the case for the basis of life (Chalmers, 1996, pp. 108-109). My suggestion that vitalism and mysterianism about consciousness make the same type of claims, that they are both epistemic claims, need not, in other words, be understood as implying that, as a normative claim, the latter ought to go the way of the former, into the intellectual dustbin of history. But the plausibility of mysterianism about consciousness is in any case not my concern in this paper.

21 Our ignorance of this truth or fact is, Stoljar notes, not just a matter of us being ignorant of whether some proposition that we understand and can formulate is true. Our predicament is worse that this: we are not able, at least at the present, to understand or formulate it (2006, locs. 1016–1021). He also points out that on his view we are not merely ignorant of some *particular* truth or set of truths, but of a *type* of non-experiential truth relevant to experience (ibid., locs. 1043–1066).

22 Hull (1974, p. 129) goes so far as to suggest that organicism, with its insistence that life is ‘an organisational property of certain material systems’, was itself a form of vitalism, just as I noted above that Wolfe does (see also Walsh, 2018). If the vital force *was* just this organisational property of living systems, rather than some imponderable ‘thing’ or ‘substance’ (much like space, time, gravity and magnetism are now understood to be properties of material bodies rather than things or substances), the metaphysical objections to vitalism evaporate. In my view Hull, like Wolfe, goes too far in claiming that organicism *is* a form of vitalism. One might just as well say that modern functionalism about the mind *just is* a form of Cartesian dualism. But he is drawing attention to the important connections between the holism of vitalism and the holism of organicism that I am also highlighting. Rather than saying organicism is a type of vitalism, I should prefer to say that vitalism (as an ignorance view) posed the questions to which organicism supplied the answers.

23 Thank you to an anonymous referee for urging me to clarify this point.

24 Mossio does argue however that, as well as this general notion, we do also require a distinctively biological principle of organisation, that can capture what is distinctive about the way living systems are organised (2024).

25 It is this modal intuition that plausibly animated vitalism understood as a metaphysical thesis. But I am suggesting that is not the only, or necessarily the best, way to understand vitalism.

26 Organicism is often presented as agreeing with mechanistic reductionism in its physicalist *metaphysics*, and merely disagreeing with it at the level of *epistemology*, i.e. concerning the sort of *explanations* that need to be given (e.g. Gilbert & Sarkar, 2000; see Chen, 2018, pp. 2–3). As it is sometimes put, organicism (along with other forms of ‘physicalist anti-reductionism’) accepts metaphysical or ontological reductionism while rejecting epistemological reductionism. I think this is a mistake. Organicists disagree with reductionists on questions of metaphysics on any meaningful sense of ‘metaphysics’. And I see no non-vacuous sense in which organicists are reductionists, either metaphysical or epistemological.

27 Thank you to two anonymous referees for pointing this out to me.

28 Whether such a view counts as a vitalist, rather than an organicist view, may be questioned however.

29 This makes the case somewhat disanalogous to our present ignorance regarding consciousness on Stoljar’s view, since on the latter, *nobody* today has any clue as to what the required type of experience-relevant nonexperiential fact or truth might be. I don’t believe this undermines the value of the analogy however; in other respects the two cases are quite similar.

30 Walsh (2018), a contemporary organicist, identifies and defends a ‘methodological’ form of vitalism, which he finds in Broad, E.S. Russell and others: the injunction to search for non-mechanistic, irreducibly biological laws and principles to account for living systems, especially organisms-as-agents (recalling what Wolfe terms ‘functional’ or ‘non-metaphysical’ vitalism; see above). This is broadly consistent with my account of the relation between organicism and vitalism on the epistemic construal, but I would express it slightly differently from Walsh. Rather than in effect *identifying* organicism with methodological vitalism, I would still want to distinguish vitalism as an ignorance claim from organicism as the claim that our ignorance has been overcome by knowledge of the principle of organisation. And while I am happy to accept that vitalism and organicism *share* the methodological commitment he describes, I think it is misleading to call this a *vitalist* methodological commitment. These might be partly verbal issues, however.

31 ‘It would be ahistorical to ridicule vitalists. When one reads the writings of one of the leading vitalists like Driesch one is forced to agree with him that many of the basic problems of biology simply cannot be solved by a philosophy as that of Descartes, in which the organism is simply considered a machine… The logic of the critique of the vitalists was impeccable’ (Mayr, 2002).

32 This applies also to Stoljar’s ignorance claim about consciousness. It clearly does not, in itself, specify a positive program for scientific research into the nature of consciousness—unless Stoljar gives us some clue as to what the nonexperiential truths relevant to experience might be like, which he doesn’t, and of course can’t, given his claim is that we are profoundly ignorant of what they might be. Still, it could potentially be useful in convincing scientists and philosophers working on consciousness that, as he puts it, ‘the nonexperiential facts are not all in’, that a different *type* of nonexperiential fact should be sought to account for consciousness, that scientific ‘business as usual’ is not going to alleviate our ignorance.

33 A way to express this is that when these physico-chemical facts were finally in, the vitalist would presumably still be unsatisfied. Something is still being left out, she would claim. The physico-chemical facts still don’t explain life fully. But when the organicists proposed organisation as the missing ingredient, in keeping with holism and anti-mechanism, it was more plausible vitalists would have been convinced that we now have a satisfactory account of life, less likely they would have thought something was being left out.
